# Current evidence for spinal X-ray use in the chiropractic profession: a narrative review

**DOI:** 10.1186/s12998-018-0217-8

**Published:** 2018-11-21

**Authors:** Hazel J Jenkins, Aron S Downie, Craig S Moore, Simon D French

**Affiliations:** 1 Department of Chiropractic, Faculty of Science and Engineering, Macquarie University, Sydney, Australia; 2 Faculty of Health, University of Technology Sydney, Sydney, Australia; 3 School of Rehabilitation Therapy, Queen’s University, Kingston, ON Canada

**Keywords:** Chiropractic, Spinal X-rays, Clinical guidelines, Appropriate use of imaging, Low back pain, Back pain, Neck pain, Imaging indications

## Abstract

The use of routine spinal X-rays within chiropractic has a contentious history. Elements of the profession advocate for the need for routine spinal X-rays to improve patient management, whereas other chiropractors advocate using spinal X-rays only when endorsed by current imaging guidelines. This review aims to summarise the current evidence for the use of spinal X-ray in chiropractic practice, with consideration of the related risks and benefits. Current evidence supports the use of spinal X-rays only in the diagnosis of trauma and spondyloarthropathy, and in the assessment of progressive spinal structural deformities such as adolescent idiopathic scoliosis. MRI is indicated to diagnose serious pathology such as cancer or infection, and to assess the need for surgical management in radiculopathy and spinal stenosis. Strong evidence demonstrates risks of imaging such as excessive radiation exposure, overdiagnosis, subsequent low-value investigation and treatment procedures, and increased costs. In most cases the potential benefits from routine imaging, including spinal X-rays, do not outweigh the potential harms. The use of spinal X-rays should not be routinely performed in chiropractic practice, and should be guided by clinical guidelines and clinician judgement.

## Background

Chiropractic has a long association with the use of spinal X-rays in clinical practice. Early X-ray technology was incorporated within chiropractic clinical examinations from 1910, with the stated purpose to visualise the alignment of spinal vertebrae and direct appropriate treatment [1, 2]. Since that time chiropractors around the globe have gained licensure for X-ray machine ownership and use. Over the last three decades the evidence-base for the diagnosis and management of spinal pain has transitioned from a static mechanical model, as visualised by X-ray, to a patient-centred model operating within a biopsychosocial context [3]. This transition, combined with the low diagnostic yield of clinically relevant radiographic findings [4], and increased awareness of associated risks has led to questioning of the routine use of imaging (including X-rays) to evaluate spinal pain [5, 6]. Current evidence-based guidelines recommend that imaging be limited predominantly to cases of suspected underlying serious pathology or trauma [7–13]. Controversy exists within the chiropractic profession, however, with some groups advocating for continued routine use of spinal X-rays within chiropractic clinical practice [14, 15]. The aim of this review is to summarise the current evidence for the use of spinal X-ray in chiropractic practice, with consideration of the related risks and benefits. The review is presented in four sections: 1) the current use of spinal X-ray imaging within chiropractic clinical practice; 2) the evidence for potential reasons for obtaining spinal X-rays within chiropractic; 3) the evidence of possible risks or limitations associated with the use of spinal X-rays; and 4) guidelines for the appropriate use of imaging in chiropractic clinical practice.

### Search strategies and study selection

PubMed and The Index of Chiropractic Literature were searched using broad search terms such as: chiropractic, spinal X-rays, adverse events, imaging risks and benefits, X-ray radiation exposure, and back and neck pain guidelines. Reference lists from relevant review articles were also searched. Guidelines and systematic reviews were selected where possible.

### Current use of spinal X-ray within chiropractic clinical practice

The proportion of patients receiving X-ray as a result of chiropractic consultation ranges from 8 to 84% [16–24]. Significant decrease in X-ray utilisation over time has been shown in some studies [16, 20, 25], whereas an increase in X-ray utilisation by chiropractors over time has also been identified [23]. A recent Australian Medicare benefits schedule review taskforce concluded that spinal X-rays continued to be overused within chiropractic clinical practice [26]. As a result, Medicare rebates for three and four-region spinal X-rays in Australia under the public health system have been withdrawn from chiropractors, while remaining for physiotherapists and osteopaths [26]. Of additional concern, only 50% of Australian chiropractors report awareness of current radiographic guidelines for low back pain [27].

Reasons given for obtaining spinal X-rays by chiropractic practitioners are varied, with many not supported by evidence of benefit. These include diagnosis of pathology or trauma; determination of treatment options; detection of contraindications to care; spinal biomechanical analysis; patient reassurance; and medicolegal reasons [27–30]. Specific drivers of these varied reasons for obtaining X-rays are unknown, although some associations have been demonstrated including lack of education, ownership of X-ray facilities, and preferred chiropractic technique modalities [27]. A number of different treatment technique modalities exist within chiropractic, some of which advocate the use of routine spinal X-rays to perform biomechanical analysis, direct appropriate treatment, and perform patient reassessment [2]. Different chiropractic educational institutions show a similar variance in their teaching of potential reasons to obtain X-rays [31], and this is reflected in an association between both rates of X-ray utilisation [17], and unorthodox use of radiography [32], with the institution of chiropractic education.

There is a growing movement in healthcare to reduce low-value care, including unnecessary and wasteful tests and procedures [33–35], spearheaded by the Choosing Wisely™ movement [36]. Choosing Wisely™ works with healthcare organisations and patient groups to develop campaigns to address overuse of tests that do not add value for patients and may cause harm. Reducing inappropriate imaging for low back pain is a prominent message in many Choosing Wisely™ campaigns for different healthcare providers, including the American Chiropractic Association and Chiropractic Australia [37–40]. This has been driven by inappropriate imaging that leads to patient harm, inefficiencies and waste in the healthcare system [36, 40, 41].

### Evidence for potential reasons for obtaining spinal X-rays within chiropractic

#### Diagnosis of pathology or trauma

Spinal X-rays are well established as a diagnostic modality to aid in the confirmation of suspected serious pathology or traumatic injury, as reflected in current imaging guidelines [7–13]. A chiropractors’ diagnosis of underlying serious pathology (i.e. cancer, infection, spondyloarthropathy) or traumatic fracture is important as clinical management would necessitate referral to medical specialities and may contraindicate manipulative therapy.

Serious pathology and traumatic injury, however, are rare causes of spinal pain. Various studies have found the incidence of serious pathology presenting as low back pain in primary care settings to be between 0.2 and 3.1% [4, 21, 42–44], and fracture to be between 0.2 and 6.6% [4, 21, 42–44]. Therefore, while these diagnoses are important to be made when the clinical circumstances exist, routine use of X-ray imaging to diagnose these conditions is not recommended due to the rarity of these presentations in clinical practice. Furthermore, recent evidence informed consensus suggests referral for MRI and blood tests, rather than X-ray, as the preferred investigation when serious pathology such as cancer or infection is suspected [45].

Spinal X-ray imaging may also be used to diagnose more benign spinal findings such as degenerative arthritis, spondylolisthesis, and transitional vertebral segments. An important consideration, however, is whether these radiographic findings are clinically important, and whether there is evidence that diagnosis by X-ray leads to a change in patient management. Many of these radiographic findings, although relatively common [21, 46, 47], show either no or weak association with symptomatology [48–52], making their clinical relevance questionable. Furthermore, there is no high quality evidence to demonstrate that patient management should be modified based on presence of benign radiographic findings that could not be determined from patient clinical history or exam alone. Current chiropractic clinical practice guidelines do not differentiate between treatment options based on the presence or absence of these benign radiographic findings [10]. Therefore, based on the evidence, the use of X-ray imaging to diagnose benign spinal findings will not improve patient outcomes or safety.

#### Determining treatment options

The use of spinal X-ray imaging may be justified where findings may result in a beneficial change to patient management. For patients presenting with conditions such as radiculopathy or spinal stenosis, imaging may be used to support the decision-making process (e.g. to determine whether conservative or surgical care is more appropriate) [7, 8, 45]. However, most cases of radiculopathy or stenosis can initially undergo a trial of conservative care [45, 53], and there is no evidence that early imaging leads to improved management beyond that informed by the clinical presentation alone [54]. Current guidelines recommend that imaging be deferred until after a trial of care, and is only indicated in cases of progressive or widespread neurological compromise [7, 8, 45]. In these cases, X-ray is not recommended and MRI is the more valuable form of imaging for assessment [8, 45].

The use of spinal X-ray imaging has been postulated to be important to help direct appropriate chiropractic management, where specific X-ray findings would lead to a change in the type of technique modality selected. However, we could find no studies assessing the impact of routine imaging on technique modality selection resulting in improved patient outcomes. While there are many different technique modalities used within chiropractic practice, there is a lack of high quality evidence to indicate which technique modalities are superior for a given condition. Furthermore, spinal X-ray has not been found to be a useful method to determine the site of spinal manipulation [55]. For usual medical care of non-specific back or neck pain, studies show no difference in treatment outcome when routine spinal X-rays have been used, compared to management without X-rays [56, 57]. Therefore, without any clear evidence of benefit of using spinal X-ray to direct treatment modality selection, clinician selection of modality should be made based on the clinical presentation, and the use of initial X-ray confirmation is not justified..

#### Screening patients for contraindications prior to care

A common reason suggested by chiropractors for spinal X-ray imaging is to screen for anomalies or serious pathology that may contraindicate treatment that were otherwise unsuspected by the clinical presentation. While some cases of serious pathology, such as cancer and infection, may not initially present with definitive symptoms, X-ray assessment at this early stage of the disease process is also likely to be negative, and is not recommended as a screening tool [58]. The development of symptoms, which would then indicate the need for imaging referral, often reflects progression of the underlying pathology, and therefore an increased likelihood of observing related imaging findings. However, even in symptomatic patients, MRI rather than X-ray is recommended as the initial imaging modality due to the higher sensitivity of MRI for the detection of pathological changes [45, 58]. Pathological causes of back and neck pain are rare [4, 42, 44], and even fewer cases would be asymptomatic, further reducing the potential benefit of routine imaging. Furthermore, imaging referral consistent with current imaging guidelines has not been shown to have an increased risk of missing serious pathology [44, 59]. Therefore, routine imaging (including spinal X-rays) for unsuspected serious pathology is not supported by evidence.

Anatomical anomalies in the upper cervical spine, such as agenesis of the dens and fusion of the occiput and atlas, have been postulated to be associated with increased upper cervical instability or neural compromise that may contraindicate manipulative therapy [46, 60]. These anomalies present with varied symptomatology, and can be difficult to clinically diagnose, thus X-ray screening has been suggested [21]. However, the contraindication of manipulative therapy for patients with these anomalies is on a theoretical basis, rather than documented clinical evidence of harm [61]. A scoping review of risks of manual treatment to the spine did not identify any reports of harm after manipulative therapy that were attributed to the presence of upper cervical anatomical anomalies [62]. Prevalence rates of upper cervical anatomical anomalies are also low (between 2.1 to 3.7% [21, 46]). The low prevalence, combined with uncertain clinical significance suggests that the use of routine X-ray to screen for congenital anomalies in asymptomatic patients is not supported by evidence.

#### Spinal biomechanical analysis

Spinal biomechanical analysis, or spinography, has long been associated with chiropractic X-ray imaging. Early chiropractic practice was focused on the correction of spinal misalignment through spinal manipulation (adjustment), with spinography used to inform this process [1]. Typically, X-ray images would be analysed to measure intersegmental rotation, tilt, or displacement and to measure spinal curvatures. Specific spinal adjustments could then be selected to correct the measured segmental or global misalignments. Additionally, post-treatment X-rays have been used as an outcome measure to assess for improvement in segmental or global spinal alignment with treatment [15].

The clinical significance of variations in spinal curvatures commonly found on X-ray imaging remains controversial. Although there is evidence that X-ray can be a reliable tool for assessing intersegmental or global spinal alignment, with good measures of inter-and intra-examiner reliability [63–65], the clinical relevance of these findings and usefulness in directing subsequent treatment selection has not been sufficiently demonstrated. In addition, alterations in X-ray spinal alignment may also reflect other factors such as variations in patient positioning during X-ray imaging, pain, or short-term muscle spasm [66], and as such may not be appropriate to inform ongoing patient management.

Some studies have reported an association between pain and variation in spinal curvatures [67–69], while others have not found significant association [70, 71] or argue that the association may be coincidental or reflective of normal variability [72, 73]. Individual study findings that associate symptomology with changes in spinal curves must be balanced by the findings from a systematic review by Christensen and Hartvigsen who found no robust evidence of association [74]. Treatment directed by biomechanical X-ray analysis of the spine has shown some evidence for a positive effect on both spinal curves and pain [75, 76], however, findings related to pain are not consistently demonstrated [77]. It is also unclear whether X-ray analysis of spinal posture is required or whether visual analysis would result in similar treatments being provided [78–81]. Therefore, it is unclear whether treatments using biomechanical X-ray analysis produce better outcomes, including any additional short and long-term cost and health benefits, compared to treatment without the use of X-ray analysis. As a result, there is currently insufficient evidence to recommend the use of routine spinal X-rays to analyse spinal biomechanics.

In contrast, X-ray analysis of structural spinal deformities is recommended to direct appropriate treatment in children or adolescents, where curve progression is of concern, or in adults with a progressive or acutely painful scoliosis or thoracic kyphosis [7]. In these cases, X-ray findings may result in alternate management, such as bracing or spinal surgery, may be necessary to prevent further deformity, or may reveal potential pathological causes of acutely painful or progressive spinal curves [82]. There is no current evidence that X-ray analysis of benign scoliotic curves in adults, or functional scoliotic curves in children or adolescents is required, or that it will improve conservative management in a significant way.

#### Patient reassurance

The use of imaging to reassure patients that they have no underlying pathology has been reported as a potential reason for imaging referral. Patients often expect imaging for the management of back pain [83], largely because they believe that it will help to diagnose their pain and direct suitable treatments [84]. However, routine use of imaging has actually been associated with a lesser sense of wellbeing [85], and lower overall health status [56]. Other strategies to reassure the patient such as education and explanation of the evidence about the use of routine imaging should be used as a first approach [8, 53, 59].

#### Medicolegal reasons

The use of imaging can be related to practitioner medicolegal concerns and the perceived risk that routine imaging reduces the risk of missing a more serious diagnosis [86, 87]. As discussed, research does not support the use of X-ray imaging as a reliable tool for the early detection of underlying serious pathology, or in screening for unsuspected anomalies. The authors are not aware of evidence where routine imaging has decreased the risk of malpractice claims made against chiropractors.

### Evidence of possible risks or limitations associated with the use of spinal X-rays

#### Radiation exposure

Radiation exposure from spinal X-rays is well recognised and quantifiable, ranging from 0.2 mSv for cervical spine X-rays, 1.5 mSv for lumbar spine X-rays, to 2.7 mSv for three-region spine X-rays [88]. These are considered to be low levels of single exposure, comparable to less than 1 year of exposure to natural background radiation [89]; however, cumulative exposure also needs to be considered, with some chiropractors’ advocating repeat spinal X-rays to monitor spinal change from the care provided [15]. The collective dose from spinal imaging is high and chiropractors’ have been shown to have a relatively high contribution to that collective dose [19], without corresponding high levels of demonstrated patient benefit.

Risks of harm from low levels of radiation exposure are difficult to calculate. Increased risk of cancer has been definitively associated with high-levels of radiation exposure in survivors of the atomic bombings of Hiroshima and Nagasaki [90]. Risk from low-levels of radiation exposure has been extrapolated using the linear no-threshold model [90]. This model assumes no safe level of radiation exposure, with a linear association between radiation exposure and risk of cancer. Epidemiological studies have associated protracted low-level radiation exposure and CT scans in childhood with increased cancer risk [91–93]; however, increased risk of cancer at low-levels of radiation exposure and the accuracy of the linear no-threshold model have not been definitively demonstrated.

Alternate models for the risk of low-levels of radiation have been postulated, including the linear threshold and hormetic models [94]. The linear threshold model incorporates a threshold below which radiation exposure is not associated with increased risk of cancer [94]. The hormetic model postulates that low levels of radiation exposure may in fact produce benefits rather than damage to tissue [94]. Evidence for these models is inconclusive [94, 95], and uncertainty remains as to the risk associated with low-level radiation exposure. In addition, even assuming these alternate models to be accurate, there is no definitive data regarding the exact threshold of radiation exposure that would be considered either beneficial or safe. There is also no way to accurately calculate past radiation exposure from natural and additional sources to account for summative levels of radiation exposure that might increase risk. Therefore, regardless of the model of risk of low levels of radiation exposure used, it is not currently possible to define a ‘safe’ level of radiation exposure, with no additional risk.

Without definitive thresholds of safe levels of radiation exposure, it should be assumed that some level of risk is associated with the use of X-rays. This risk is considered under the precautionary principle to ‘First do no harm’ [96], and is recognised by practice standards and radiation protection principles advocating the ‘As Low As Reasonably Achievable’ (ALARA) principle [97]. Whether X-rays are taken, which X-ray series are requested, and the technique used to perform the X-ray are all important considerations to ensure that radiation exposure is as low as possible [97].

The risk from radiation exposure should not be considered a barrier to requesting imaging where it is clinically justifiable. Even using the linear no-threshold model, lifetime risk of cancer from a single X-ray is considered to be minimal in the neck (1/1000000–1/100000) and very low in the spine (1/100000–1/10000) [95], and, therefore, should not be a reason to limit the use of X-ray when clinically indicated, as recommended in evidence based guidelines [7–13].

#### Overdiagnosis

Spinal X-rays may lead to the detection of radiographic findings of uncertain clinical significance, leading to unnecessary diagnosis (overdiagnosis). X-ray findings, such as osteophytes, reduced disc height, spondylolisthesis, transitional segments, and other anatomical anomalies are common [21, 46–48, 52], but show poor correlation with clinical symptoms [48–51, 98, 99]. Brinjikji et al. found a high prevalence of disc degeneration in asymptomatic individuals, ranging from 37% of 20-year-olds to 96% of 80-year-olds [52]. Carragee et al. found that 84% of new low back pain presentations had unchanged or improved imaging findings when compared to baseline images taken when asymptomatic [100], and Panagopoulos et al. found similar radiographic findings between low back pain patients and healthy controls, with similar changes to these findings seen over time [101]. Furthermore, beneficial changes to conservative management of the patient is unlikely when these X-ray findings are present, with no robust evidence that early imaging improves clinical outcomes [56, 57, 85, 102–107].

Inconclusive X-ray findings such as suspicion of pathology may lead to more complex investigation and unnecessary worry for the patient before the diagnosis (or lack thereof) is confirmed. Wnuk et al. found that of 78% of cases of suspected cancer or infection on imaging were found to be false positive results [42]. For patients without indicators of serious pathology, the increase in information available from X-ray confers little additional benefit to patient health, but may unnecessarily increase patient concern and thus contribute to low value care [33, 34].

Overdiagnosis may create unwarranted concern for the patient [108, 109] and a misguided belief in a pathoanatomical cause to their pain [99]. Patients may believe that their pain will not improve until the imaging findings have resolved, which may increase the risk of developing chronic pain [53, 99, 103]. Overdiagnosis may also contribute to fear-avoidance behaviours, where patients are less likely to follow management advice (e.g. maintaining exercise and physical activity) for fear of further damage [8, 53]. Early imaging of the low back has been associated with resultant increased disability [103, 105], a lesser sense of well-being [85], and lower health status [56].

#### Missed diagnosis

Early use of spinal X-rays may lead to false negative results (type II error), due to inability to detect early pathological change, or the use of imaging modalities with poor diagnostic utility based on convenience. This may lead to false reassurance of absence of pathology, and may delay the use of appropriate imaging when the disease is sufficiently progressed to demonstrate imaging findings. Although X-ray is often used to screen for pathology, it does not have high sensitivity for early detection of pathology [58, 110]. For example, there needs to be a minimum of between 30 to 50% loss in bone mass before pathology is often detectible on X-ray [111], and overlapping anatomy may also obscure pathological changes to further limit radiographic diagnosis. Poor radiographic technique or reporting errors may also lead to false negative results [112].

#### Waste

Inappropriate use of spinal X-rays and other imaging techniques may lead to waste in the form of unnecessary invasive diagnostic procedures and subsequent treatment, increased waiting time for people who are in need of more appropriate imaging, excessive costs and poor utilisation of human resources [103, 113–117].

The use of spinal X-ray when not indicated leads to increased financial cost to the health care system, the patient, and the population. Financial costs are related to that of the spinal X-ray itself, and downstream costs due to increased healthcare utilisation, poorer patient outcome, poorer productivity, or increased disease burden [53, 99, 118, 119]. Early imaging has been associated with a greater use of medical care and associated costs [53]. Webster et al. found that early imaging was associated with an increase in use of medical services, with costs between $7643 to $13,816 higher per episode than the group without imaging [103]. The authors hypothesised that this increase in costs may have been the result of treatment or investigation of clinically insignificant radiographic findings found on the initial imaging [103]. Similarly, Jarvik et al. found that total costs were between 27 to 30% higher in early imaging groups compared to no imaging groups [106]. Depending on the healthcare system, patients may not have substantial out of pocket expenses when referred for imaging, however, the expense to the public health system is high, and reflected in increased indirect costs to the population.

### Guidelines for the appropriate use of imaging

Most cases of acute spinal pain improve within the first 4 weeks [120–122], with imaging discouraged within this time period to allow for natural recovery [8, 53]. The decision to use imaging to manage patients with back and neck pain is a balance between the consideration of potential risks and benefits for each patient. To facilitate this process, guidelines for the chiropractic profession [7, 10], and for primary medical care [8, 9, 11–13] have been produced to help guide clinicians in the decision-making process. These guidelines have been based on the available evidence and therefore recommend that diagnostic imaging is used only when there is clinical suspicion of serious pathology or when imaging findings are likely to lead to a beneficial change in management, improvement in patient outcome, or decrease in patient harm. Failure of a patient to respond to care over a four to 6 week time period may indicate the need for imaging; however, imaging should still only be undertaken when there is a suspected serious pathological cause of the patient’s pain, or it is anticipated that a significant change in management will result [7, 8]. A summary of current imaging guidelines is presented in Table 1.

**Table 1:** Summary of current evidence based guideline recommendations for diagnostic imaging of the spine for chiropractors [7, 8, 13, 45, 82, 124, 129, 130]

Clinical suspicion	Alerting clinical features^a^	Recommended imaging, referral or clinical action
Spinal fracture (cervical)	Canadian Cervical Spine Rule (C-Spine Rule) [13] History of cervical trauma and any one of (assessment to be performed in order): 1. Presence of at least one high risk factor (age of 65 years or above; dangerous mechanism of injury (e.g. fall of greater than 5 stairs); extremity paraesthesias) 2. Absence of all low risk factors (simple rear-end motor vehicle accident; sitting position at presentation; ambulatory at any time post trauma; delayed onset of neck pain; absence of midline c-spine tenderness) 3. Inability to actively rotate neck 45 degrees left and right	• Cervical X-ray: AP, APOM, and Lateral • May also require CT or MRI for complete assessment
Spinal fracture (other region)	Spinal pain after recent history of significant trauma with multiple risk factors: • Older age (above 65 years for women, above 75 years for men) • History of osteoporosis • Prolonged corticosteroid use • Severe trauma • Contusion or abrasion	• X-ray • If negative X-ray result and strong clinical suspicion consider MRI
Cancer	Major risk factors for cancer: • New onset of spinal pain with history of cancer • Multiple risk factors or strong clinical suspicion of cancer (breast, lung, and prostate are the most common primary sites) Weaker risk factors for cancer: • Age greater than 60 years • Unexplained weight loss • Pain with rest or at night • Failure to improve after one month with conservative care	Major risk factors present: • Immediate imaging: MRI (if MRI unavailable, X-ray suitable) • Blood tests No major risk factors present: • Trial of appropriate conservative therapy prior to further diagnostic workup
Infection	New onset of spinal pain with risk factors of infection: • Fever or chills • History of infection • History of intravenous drug use • Recent spinal surgical or investigative procedure • Pain with rest or at night	• MRI and blood tests • Specialist referral^b^
Spondyloarthropathy	Chronic pain (greater than 3 months) with risk factors of spondyloarthropathy: • Younger age at onset (less than 40 years) • Insidious onset • Improves with exercise • Alternating buttock pain • Pain at night • Positive family history • Extremity articular symptoms • Improvement with non-steroidal anti-inflammatory drugs • Extra-articular symptoms (I.e. psoriasis, inflammatory bowel disease, uveitis)	Strong clinical suspicion: • X-ray and blood tests • If negative X-ray result and strong clinical suspicion or positive blood tests consider MRI • Specialist referral^b^ Lower clinical suspicion: • Trial of appropriate conservative therapy prior to further diagnostic workup
Radiculopathy	Back or neck pain with leg or arm pain, sensory loss, weakness, or decreased reflexes	Single-level radiculopathy: • Trial of appropriate conservative therapy prior to further diagnostic workup Multi-level or progressive neurological symptoms (especially motor or reflex deficits), or surgical candidates: • MRI • Specialist referral^b^
Lumbar spinal canal stenosis	Risk factors of neurogenic claudication: • Older age • Buttock, thigh or leg pain • Worse with walking/standing • Relieved by sitting or flexed postures	Non-surgical candidates: • Trial of appropriate conservative therapy prior to diagnostic workup Surgical candidates: • MRI • Specialist referral^b^
Spinal cord compression	Risk factors for cervical myelopathy: • Neck pain with multi-level, progressive upper limb neurological symptoms (especially motor or reflex deficits) • Older age • Increased lower limb reflexes Risk factors for cauda equina syndrome: • Multi-level, progressive lower limb neurological symptoms (especially motor or reflex deficits) • New bowel or bladder dysfunction • Saddle anaesthesia	Acute/severe symptoms: • Emergency referral, no prior imaging Chronic/less severe symptoms: • MRI • Specialist referral^b^
Arterial dissection, stenosis, or aneurysm	Cervical spine risk factors: • Severe, persistent or unusual neck pain or headache • Cranial or upper limb neurologic symptoms Thoracic spine risk factors: • Severe chest or back pain • Hypotension • Absent distal pulses Lumbar spine risk factors: • Severe abdominal, back, or groin pain • Hypotension • Absent distal pulses	Acute/severe symptoms: • Emergency referral, no prior imaging Chronic/less severe symptoms: • Ultrasound or MRI • Specialist referral^b^
Osteoporosis	Major risk factors: • History of fracture as a result of minimal trauma • History of prolonged corticosteroid use • Older age (greater than 65 years in females, greater than 75 years in males) • Premature menopause in females • Hypogonadism in males • Predisposing condition (I.e. rheumatoid arthritis, hyperthyroidism, hyperparathyroidism, chronic kidney or liver disease, coeliac disease); Weaker risk factors: • Parental history • Low physical activity • Low body weight • Poor nutrition • Poor balance • Frequent falls	• DXA^c^ scan of spine and proximal femur
Progressive spinal structural deformity	Child or adolescent: • Rigid coronal or sagittal curvature • Positive Adam’s test • Rib humping Adult: • Rigid coronal or sagittal curvature with either acute presentation of curvature, or recent progression of curve	• X-ray • Specialist referral for identified underlying pathology or large cobb angle (> 25 degrees)

^a^Single risk factors are usually not sufficient to indicate imaging referral. Clinical suspicion of the condition must also exist

^b^It may be appropriate to defer imaging referral until specialist review

^c^Dual-energy X-ray absorptiometry

Alternate X-ray guidelines for the chiropractic profession have also been proposed [123]. These guidelines have not been considered in the summary provided in Table 1 because: 1) they make the initial assumption that spinal X-rays are required for a chiropractor to provide optimal management of the patient; 2) all available high quality evidence and peer-reviewed imaging guidelines do not support the routine use of spinal imaging for spinal conditions; 3) to the best of our knowledge, the guidelines in question have not been published in a peer-reviewed journal; and 4) the guidelines do not adequately consider the well-established evidence for the potential risks of spinal X-rays, as presented in this and other review papers [8, 45, 53].

Determining which patients have sufficient clinical suspicion of serious pathology to warrant imaging can be challenging. Specific signs and symptoms associated with pathology (“red flags”) have been used in the past to help guide the selection of patients requiring imaging, however, the diagnostic accuracy of most red flags is poor [124–126]. For example, the probability of finding a spinal malignancy or spinal fracture is only modestly higher even when multiple red flags are present [124]. In addition, up to 80% of patients presenting to primary care have at least one red flag [4], limiting their usefulness in selecting specific patients for imaging. Indeed, the use of a single red flag to guide imaging has been associated with increased referral [127, 128]. As presented in Table 1, more recent guidelines [9, 45] base the decision to refer for imaging on the clinical suspicion of the referring practitioner, and provide a list of alerting clinical features that may indicate increased likelihood of serious underlying pathology. When there is lower clinical suspicion of serious underlying pathology, a strategy of watchful waiting is recommended, where a trial of appropriate conservative management is initiated and patient symptoms are monitored for progression or lack of resolution which may indicate the need for imaging [8].

A continuing question to be addressed is the appropriate rate of X-ray utilisation within the chiropractic profession, and whether this should be different to other primary healthcare professions managing spinal pain. This is an important topic for future research and cannot be answered completely from the current peer-reviewed literature. Serious pathology as the cause of LBP is rare, estimated at less than 5% of presentations [4]. As the diagnosis of serious pathology remains the main indicator for X-ray imaging, utilisation rates should also be low. At present appropriate utilisation rates cannot be precisely identified due to the unknown sensitivity and specificity of tests to indicate when imaging may be indicated. To date there is no evidence that X-rays should be used in different clinical scenarios for the chiropractic profession when compared to other primary healthcare professions such as medicine, physiotherapy, and osteopathy, and clinical guidelines across the different professions contain similar recommendations [7, 8, 10, 11]. Therefore, it is likely that X-ray utilisation rates across all primary care professions managing spinal pain should be similar.

Clinical guidelines are designed to complement, rather than replace, clinician decision-making. They are designed to provide a summary of the available evidence to indicate when it is likely that the benefits of referring for imaging outweigh the possible risks. While not all patient presentations will fit the guidelines specifically, and clinical judgement should always be used in the decision-making process, clinical judgement should be informed by the recommendations within clinical practice guidelines.

## Conclusion

The use of spinal X-rays in chiropractic has been controversial, with benefits for the use of routine spinal X-rays being proposed by some elements of the profession. However, evidence of these postulated benefits is limited or non-existent. There is strong evidence to demonstrate potential harms associated with spinal X-rays including increased ionising radiation exposure, overdiagnosis, subsequent low-value investigation and treatment procedures, and increased unnecessary costs. Therefore, in the vast majority of cases who present to chiropractors, the potential benefit from spinal X-rays does not outweigh the potential harms. Spinal X-rays should not be performed as a routine part of chiropractic practice, and the decision to perform diagnostic imaging should be informed by evidence based clinical practice guidelines and clinician judgement.
